# Draft Genome Sequence of Multidrug Resistant Klebsiella pneumoniae Strain C43 Isolated from Environmental Water Sample

**DOI:** 10.1128/mra.00252-22

**Published:** 2022-08-22

**Authors:** Soon Keong Wee, Eric Peng Huat Yap

**Affiliations:** a Lee Kong Chian School of Medicine, Nanyang Technological University, Singapore; Loyola University Chicago

## Abstract

Here, we report the genome of ESBL-producing Klebsiella pneumoniae strain C43, which was isolated from an environmental water sample. The genome is 5,614,412 bp in size with GC content of 56.86% with multidrug antimicrobial resistance genes and several metal resistance gene operons.

## ANNOUNCEMENT

Klebsiella pneumoniae is a Gram-negative Enterobacteriaceae capable of causing urinary tract infection, pneumonia, and wound or soft tissue infections (1). Apart from being a hospital-acquired pathogen, K. pneumoniae can cause a range of community-acquired infections and be transmitted through food, animal, and environmental sources. Multidrug resistance (MDR) in K. pneumoniae can be disseminated through global MDR lineages, acquisition of plasmids, and mobilization of antimicrobial resistance genes (1). We report the draft genomic sequence of an environmental Klebsiella pneumoniae strain C43 that is resistant to multiple antimicrobials.

Klebsiella pneumoniae strain C43 was isolated from water of an urban drain near Singapore in 2017. The isolate was enriched overnight in lysogeny broth (LB) at 37°C with aeration, and subsequently plated and subcultured on LB agar. Antibiotic susceptibility testing was performed by disk diffusion in accordance with Clinical and Laboratory Standards Institute (CLSI) Standards (2). The cells were grown in LB broth for bacterial DNA extraction using Qiagen DNA Mini kit following manufacturer’s instructions and sequenced with Nextera XT DNA library preparation kit (Illumina, USA). A total of 1,594,314 sequencing reads from two Illumina MiSeq runs using 250-bp paired-end and 300-bp paired-end sequencing were obtained. The FASTQ reads were combined for analysis and assembly. The genome was assembled *de novo* using Unicycler Galaxy version 0.4.6.0 (3), and 298 contigs (45-fold coverage depth, *N*_50_ of 61,998 bp) were obtained. Genome quality assessment with CheckM revealed near (98.42%) completeness and low (2.55%) contamination against Klebsiella pneumoniae (4). Annotation was performed using the NCBI Prokaryotic Genome Annotation Pipeline 4.9 (5). Species identification was confirmed using average nucleotide identity (ANI) calculator (6). Multilocus sequencing typing (MLST), strict core genome MLST (scgMLST), and *wzi* gene capsular polysaccharide characterization (K typing) were carried out using BIGSdb database v1.22.3 (7). Presence of clustered regularly interspaced short palindromic repeat (CRISPR) sequences was predicted using CRISPRCasFinder (8). Antimicrobial and metal resistance genes were predicted using NCBI AMRFinderPlus 3.0 (9). In-silico detection of plasmids was done using PlasmidFinder 2.0 (10), and prophage prediction was carried out using PHASTER (11). Default parameters were used for all software tools.

Klebsiella pneumoniae strain C43 has a genome size of 5,614,412 bp with GC content of 56.86%. The draft assembly contains 5,514 protein-coding genes, 152 pseudo-genes, 72 tRNAs, 8 rRNAs, and 10 ncRNAs. An ANI value of 99.24% was calculated using K. pneumoniae *subsp. pneumoniae* strain HS11286 (RefSeq accession number NC_016845.1) as the reference genome. Three incomplete prophage regions (25.4 kb, 23.5 kb and 11.7 kb) and a CRISPR array with 51 spacers and cas type I-E proteins were predicted. The virulence factors of K. pneumoniae strain C43 include *mrkABCDFIJ* Type 3 fimbriae for cell adherence and biofilm formation and *hsp20* heat shock protein. K. pneumoniae strain C43 belongs to ST432, scgST108 with K39 capsule type and *wzi* 39 allele. It harbors 23 antimicrobial resistance genes (ARGs) against 10 classes of antimicrobials as well as 24 metal resistance genes against 5 metals (Table 1). It is phenotypically susceptible to amikacin and meropenem; intermediate resistant to piperacillin-tazobactam, doripenem, and imipenem; and resistant to ampicillin/sulbactam, ceftazidime, cefotaxime, gentamicin, tetracycline, ciprofloxacin, and trimethoprim/sulfamethoxazole antibiotics (Table 1). The presence of multidrug resistant K. pneumoniae strain C43 in the aquatic environment suggests the possibility of a nonhospital reservoir for the transmission of antimicrobial and metal resistance genes. It poses a risk of community-acquired infection and dissemination into other environmental reservoirs.

**TABLE 1:** List of antimicrobial resistance genes and metal resistance genes present in Klebsiella pneumoniae C43 and its antibiotics susceptibility phenotype

Class	Genes	Phenotype^*b*^
Antimicrobial resistance		
Aminoglycoside	^*a*^*aph*(6)*-Id*, *aaph(3″)-Ib*, *aadA1*, *aph*(4)*-Ia*, *aac*(3)*-IV*, *aadA2*, *aph(3’)-Ia*	S: amikacin R: gentamicin
Beta-Lactam	*bla*_DHA-1_, ^*a*^*bla*_SHV-12_	S: meropenem I: piperacillin-tazobactam, doripenem, imipenem R: ampicillin/sulbactam, ceftazidime, cefotaxime
Fosfomycin	^*a*^ *fosA*	Not tested
Macrolide	*mph*(A)	Not tested
Phenicol	*floR*	Not tested
Quaternary ammonium	*qacE*, *qacL*	Not tested
Quinolone	^*a*^*oqxB19*, ^*a*^*oqxA10*, *qnrB4*, *qnrS1*	R: ciprofloxacin
Sulfonamide	*sul1*, *sul3*	R: trimethoprim/sulfamethoxazole
Tetracycline	*tet*(A)	R: tetracycline
Trimethoprim	*drfA12*, *dfrA1*	R: trimethoprim/sulfamethoxazole
Metal resistance		
Arsenic	*arsA*, *arsD*	Not tested
Copper	*pcoB*, *pcoC*, *pcoD*, *pcoR*, *pcoS*	Not tested
Silver	*silS*, *silR*, *silC*, *silF*, *silB*, *silA*, *silE*	Not tested
Mercury	*merR*, *merT*, *merP*, *merF*, *merD*, *merE*	Not tested
Tellurium	*terE*, *terD*, *terC*, *terB*	Not tested

a Genes predicted to be chromosomal.

b S, susceptible; I, intermediate resistant; R, resistant.

### Data availability.

This Whole Genome Shotgun project has been deposited in DDBJ/ENA/GenBank under the accession number VSRK00000000. The version described in this paper is the first version (VSRK01000000). The draft genome has been deposited under GenBank Assembly accession number GCA_008107405.1, BioSample number SAMN12612695, and BioProject number PRJNA561253. The raw reads are available in the Sequence Read Archive (SRA) under accession SRR10007726 and SRR10007727.
